# Supplementary material for: PhenGenVar: A User-Friendly Genetic Variant Detection and Visualization Tool for Precision Medicine
Source: J Pers Med. 2022 Jun 12;12(6):959. doi: 10.3390/jpm12060959 (PMC9224645; doi:10.3390/jpm12060959)
Supplement: Supplementary file 1 [file jpm-12-00959-s001.zip › PhenGenVar_Exom_Browser manual.pdf]

PhenGenVar Exome Browser Manual

The PhenGenVar Exome browser can effectively identify the disease-gene-variant relationship from a large genome data pool. From the Human Phenotype Ontology (HPO) database, in particular, the browser can automatically determine the genetic areas related to a specific phenotype or disease, and provides a top-down search method which facilitates variation analysis of each genetic area. Figure 1 exhibits the main page of the PhenGenVar Exome browser. Typically, the main page consists of 9 areas whose functions have been listed in Table 1.

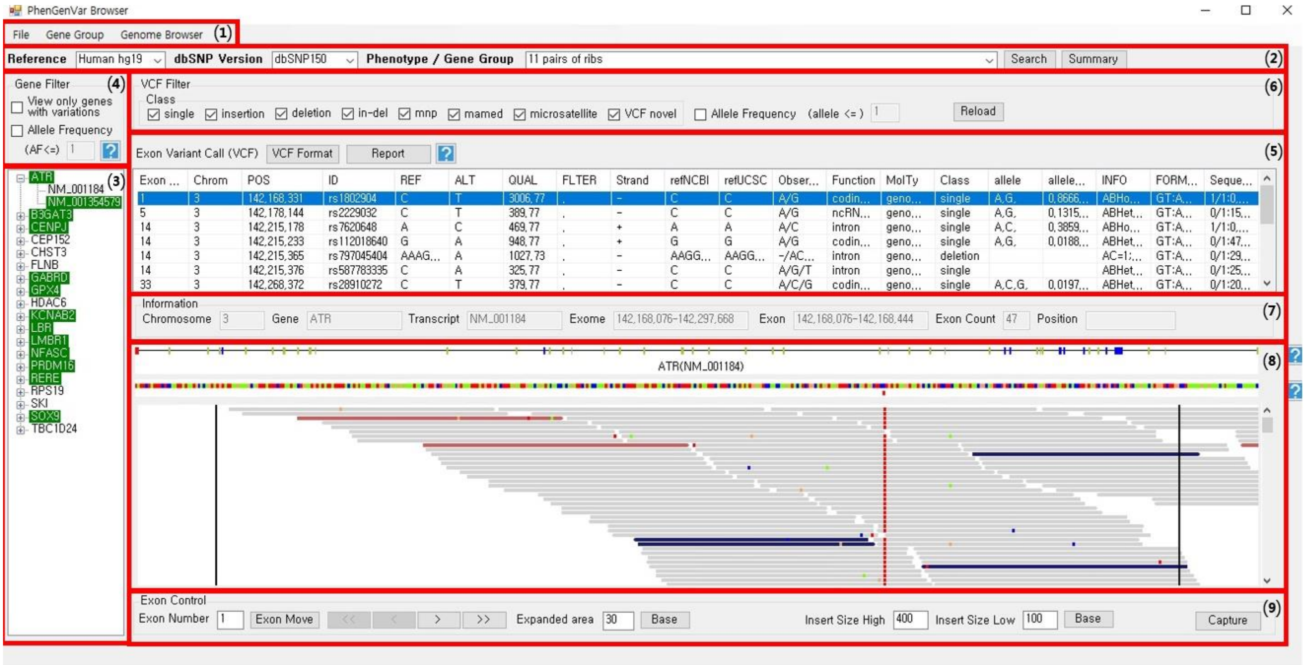

Figure 1. Main Page of PhenGenVar Exome Browser

<Table 1> PhenGenVar Exome Browser's Page Contents & their Functions

No.	Contents	Function
(1)	Menu Bar	Shows the menu.
(2)	Control Panel	Controls the BAM/VCF file analysis.
(3)	Gene/Transcript View Panel	Outputs a Gene/Transcript List.
(4)	Gene Filter Panel	Sets up the filtering conditions of Gene /Transcript List to be seen in the Gene/Transcript View Panel.
(5)	Exon Variant Call Panel	Outputs the variant information in exon area that belongs to a selected transcript.
(6)	VCF Filter Panel	Sets up the filtering conditions of variants that belong to a selected transcript.
(7)	Information Panel	Outputs information related to the presently selected variant.
(8)	Main Exon View Panel	Presents the visualization of read alignment results and

		variant manifestation by the exon unit for a selected transcript.
(9)	Exon View Control Panel	Controls the Main Exon View Panel.

(1) Menu Bar

The Menu Bar consists of File, Gene Group, and Genome Browser (See Figure 2).

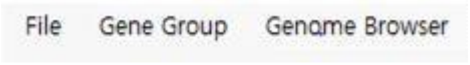

Figure 2. Menu Bar Organization

① File menu

The File menu deals with opening files in the PhenGenVar Exome browser. The menu has two sub-menus, viz. BAM file open and VCF file open (See Figure 3). The read alignment result provided by a user is input in the BAM file format, while information on genetic structural variation is input in the VCF file format.

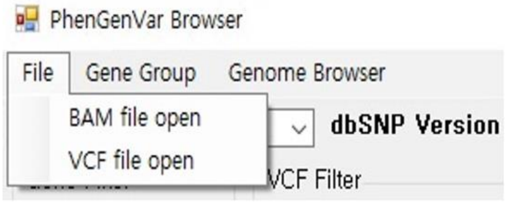

Figure 3.File Menu Organization

▶ BAM file open: A BAM file should be input to analyze read alignment result. To retrieve a BAM file, select BAM file open from the File menu. Upon its selection, a window for file selection opens up. Select a BAM file and click 'Open' to open the BAM file. To analyze the alignment result, the browser refers to a BAI file, the index file of the BAM file. Therefore, a BAM file and its BAM BAI file should be in the same folder. In the absence of a BAI file, the browser is unable to open the corresponding BAM file, and the program is not executable.

▶ VCF file open: To analyze genetic structural variation results, a VCF file should be provided. To retrieve a VCF file, select 'VCF file open' from the File menu. Upon its selection, the browser opens a window for file selection. Select a VCF file and click 'Open' to retrieve the VCF file in the browser. The browser refers to an IDX file, the index file of the VCF file. Therefore, a VCF file and its VCF IDX file should be in the same folder. In the absence of an IDX file, the browser is unable to open the corresponding VCF file.

② Gene Group menu

The Gene Group menu provides the functions to create/edit/delete a user-defined gene group. List of gene in a given gene group also can be retrieved from the public database such as Human Phenotype Ontology (HPO) or Kyoto Encyclopedia of Genes and Genomes (KEGG). The PhenGenVar Exome browser provides the gene list registered in the HPO. The list can be retrieved using Control Panel. When the Gene Group menu is executed, a dialogue box for gene group management is displayed (See Figure 4). The dialogue box consists of two list views and three buttons. Of the two list views, the Group Name

list view is on the left side of the box and outputs the gene group names created by a user previously. The Gene Name List View on the right side shows the gene symbols registered in the name of a selected group. New, Edit and Delete buttons are in the lower part of the box with the following functions.

▶ New Button: When the New Button is clicked, a dialogue box pops up which allows to add a new gene group (See Figure 5). Enter a name of the gene group to create the group in the group name list view. Next, enter gene symbols in the list view at the bottom and click OK to create a gene group with a new name. Make sure that every gene symbol input is HGNC (HUGO Gene Nomenclature Committee) official symbol.

▶ Edit Button: Select a random group from the Group Name list view under the Gene Group menus and click Edit to change the gene symbol associated with the selected group (See Figure 6). Delete a gene symbol or register a new gene symbol; then click OK to register the change for the gene group of interest.

▶ Delete Button: To delete any gene group, select the group from the Group Name list view in the dialogue box for the Gene Group menu and click Delete. Then, click OK to confirm the deletion. Such deletion is not restorable.

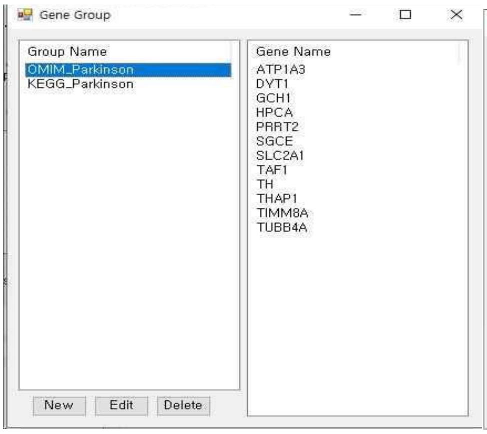

Figure 4. Dialogue Box for Gene Group Management

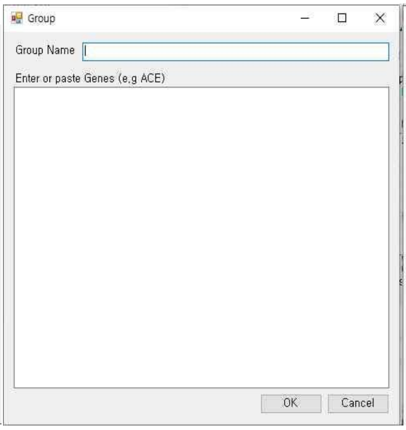

Figure 5. Dialogue Box for Gene Group Addition

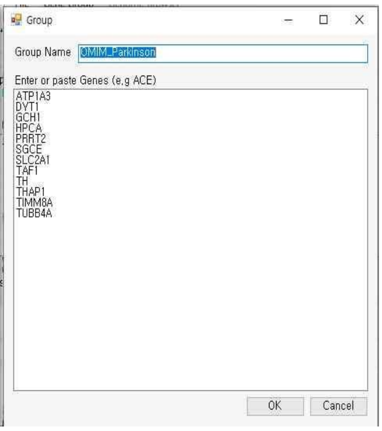

Figure 6. Dialogue Box for Gene Group Edition

③ Genome Browser menu

This menu is to call the Genome Browser for the close browsing the variant calling results based on read alignment. A BAM file provided by a user should be loaded before calling the Genome Browser. As mentioned earlier, a user can open a BAM file using the File menu. A VCF file including information on genetic structural variation can be entered selectively. If a VCF file is input, a user can gain more detailed analysis of variation information (See the Genome Browser Manual).

(2) Control Panel

The Control Panel is to select data for BAM/VCF file analysis and consists of 5 areas (See Figure 7). Table 2 exhibits the functions of each of the 5 areas.

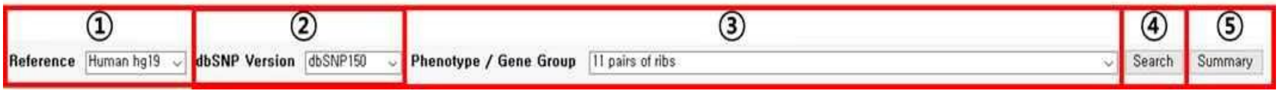

Figure 7. Five Areas of the Control Panel

<Table 2> Control Panel's Page Contents & their Functions

No.	Contents	Function
①	Reference Human hg19<br>Reference build Combo Box	Selects a reference sequence version.
②	dbSNP Version dbSNP150<br>dbSNP Version Combo Box	Selects a dbSNP version.
③	Phenotype / Gene Group 11 pairs of ribs<br>Phenotype/Gene_Group Combo Box	Selects a Gene_Group or Phenotype to analyze.
④	Search<br>Search Button	Calls the Gene Set of a selected Phenotype or Gene_Group.
⑤	Summary<br>Summary Button	Outputs the summary of variants detected in the Gene Set of a Phenotype or Gene_Group of interest.

① Reference build Combo Box

The Reference build Combo Box is used to select a reference sequence version necessary for analysis. The current browser supports the reference sequence versions of Human hg_19 and Human hg_38.

② dbSNP Version Combo Box

The dbSNP Version Combo Box is utilized to select a dbSNP version necessary for analysis. Different dbSNP versions are provided following a selected reference sequence. Table 3 shows the available dbSNP versions according to reference sequences.

<Table 3> Available dbSNP versions according to reference sequence

Reference sequence	dbSNP version
Human hg_19	SNP_138, SNP_141, SNP_142, SNP_144, SNP_146, SNP_147, SNP_150, SNP_151
Human hg_38	SNP_141, SNP_142, SNP_144, SNP_146, SNP_147, SNP_150, SNP_151

③ Phenotype/Gene_Group Combo Box

The Phenotype/Gene_Group area can be used to enter/select a phenotype or gene_group that a user intends to analyze. To search a phenotype, if a keyword is (partially) entered, word completion function is activated, and a list of keyword-related phenotypes is produced based on the HPO database. A user can select the phenotype of interest from the list. For Gene_Group, if the gene_group name of a search object is (partially) keyed in, word completion function is activated, and a user can select a registered

gene_group.

④ Search Button

The Search button is utilized to retrieve a gene set included in a gene_group or gene set (registered as a related gene set with the HPO) related to an entered/selected phenotype in the Phenotype/Gene_Group area. These gene sets are displayed in the Gene/Transcript View area. Moreover, if a user enters new filtering conditions using the Gene Filter Panel and intends to retrieve the changed gene set in the Gene/Transcript View area, the user has to click the 'Search' button again.

⑤ Summary Button

The Summary button is utilized to summarize and output the statistics of variant information (in a VCF file) entered by a user. The statistics are on a gene set of a phenotype or gene_group selected by a user. The summary is provided in the unit of a gene, as shown in Table 4. To acquire such statistics, click the 'Summary' button, and a dialogue box containing the statistical output pops up (See Figure 8). Click the 'Save' button in the statistical output window to save the output page in a tsv file with tabs for separation.

<Table 4> Details of Statistical Output through the Summary Button

Heading	Description
Gene Name	The name of a gene
Number of SNPs	Number of all SNPs called from the gene
Number of dbSNPs	Number of SNPs registered with dbSNP among all SNPs called from the gene
Number of Noble SNPs	Number of SNPs not registered with dbSNP among all SNPs called from the gene
Number of Indels	Number of all Indels called from the gene
Number of dbSNP_Indels	Number of Indels registered with dbSNP among all Indels called from the gene
Number of Noble_Indels	Number of Indels not registered with dbSNP among all Indels called from the gene
Number of multiallelic sites	Number of all multiallelic sites called from the gene
Number of multiallelic SNP sites	Number of SNPs among all multiallelic sites called from the gene

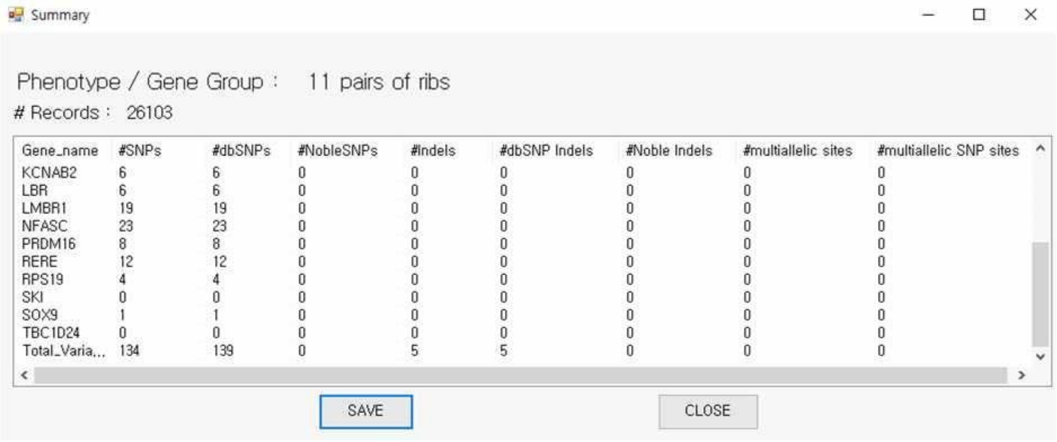

Figure 8. Statistical Output Page

(3) Gene/Transcript View Panel

In the Gene/Transcript View area, the gene set of a phenotype/gene_group selected by a user is printed out together with its transcript data (See the lower part of Figure 9). In the event of a report on variant calling from the Gene/Transcript, the background color of the name of that corresponding Gene/Transcript is displayed as green, whereas, in the event of no report on variant calling the background appears white. The variant information is extracted from the VCF file entered by the user. Every gene output in the Gene/Transcript View area employs the Official Symbols approved by the HGNC Synonyms. A user can double click a random gene output in the Gene/Transcript View area to obtain the transcript data of the gene in a child node. If a random transcript output in a child node is double-clicked, the Browser's Exon Variant Call Panel opens and the Information areas are activated to provide the detailed analysis of the exon/intron structure, variant information, of the transcript of interest.

(4) Gene Filter Panel

Filtering conditions can be added to the Gene/Transcript set output in the Gene/Transcript View Panel (See the upper part of Figure 9). The two filtering conditions shown in Table 5 are supported. Each filtering condition is activated using the checkmark button. A user can use "View only genes with variations" to set the output as only the Genes/Transcripts with variation report in the Gene/Transcript View area. A user can also utilize the "Allele Frequency" option to impose the restriction that only the Genes/Transcripts that have a variation meeting the related conditions would be displayed as the output. When the Allele Frequency filter is not ticked, the default output contains all the genes/transcripts with allele frequency not higher than 1. On the other hand, if the filter is ticked and the allele frequency value is set, the Genes/Transcripts are filtered accordingly and produced as output. Users need to make sure to click the Search button after setting such filtering conditions to reflect the filtering results in the Gene/Transcript View area.

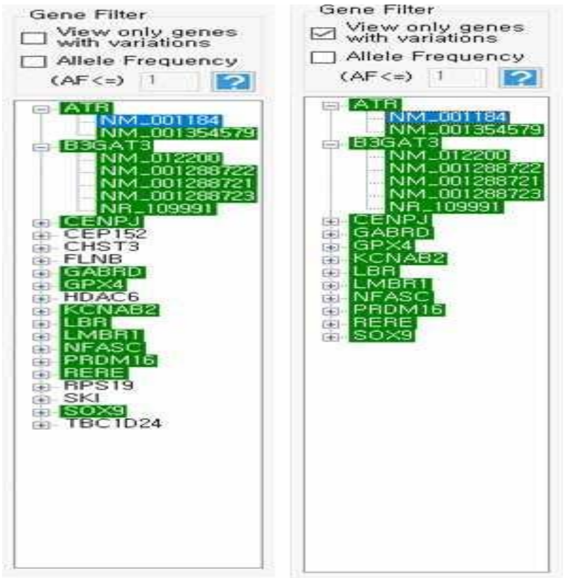

Figure 9. The Gene Filter Panel and Gene/Transcript View Panel

<Table 5> Gene Filter Functions

Filtering condition	Function
View only genes with variations	Outputs only the Genes/Transcripts with variation.
Allele Frequency	Outputs only the Genes/Transcripts having a variation meeting the allele frequency condition.

(5) Exon Variant Call Panel

The Exon Variant Call Panel produces the variant information extracted from the transcript selected by a user in the Gene/Transcript View area (See panel (1) in the lower part of Figure 10). Variant information is created based on a VCF file input by a user. Of the variants extracted from a VCF file, those with rsID are linked to the dbSNP database, and part of the dbSNP information is output together with the variant information. In the absence of rsID, only the information included in the VCF file is displayed.

Select and click a random variant to generate an exon area including the position of the selected variant in the Main Exon View Panel. Information on the related exon area will be displayed in the Information Panel. Select a variant and double click with a mouse to call a Genome Browser centering at the position of the related variant and use it for a detailed analysis of that area.

Click the ‘VCF Format’ button in the upper part to open a window for the VCF Format information check. FORMAT information and INFO are output together with the filtering information entered at the time of VCF file creation (See Figure 11). Click the ‘Report’ button to open a dialogue box to save the output results in the Exon Variant Call Panel. Click ‘Save’ within the box to save the results as a tsv file.

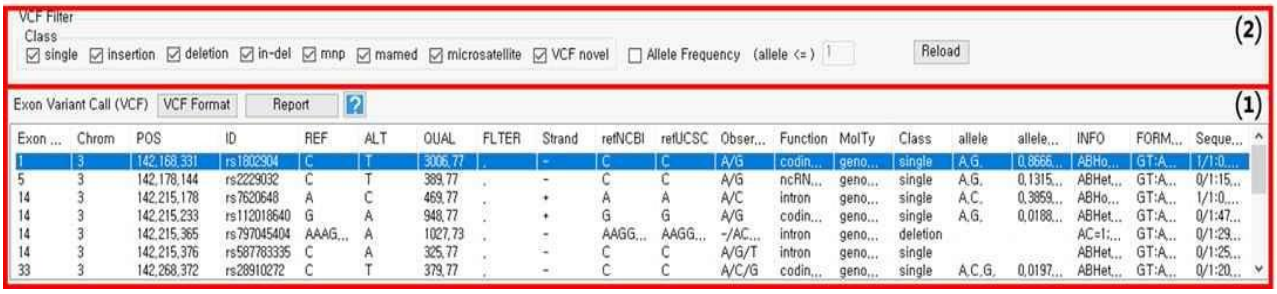

Figure 10. The Exon Variant Call Panel and VCF Filter panel

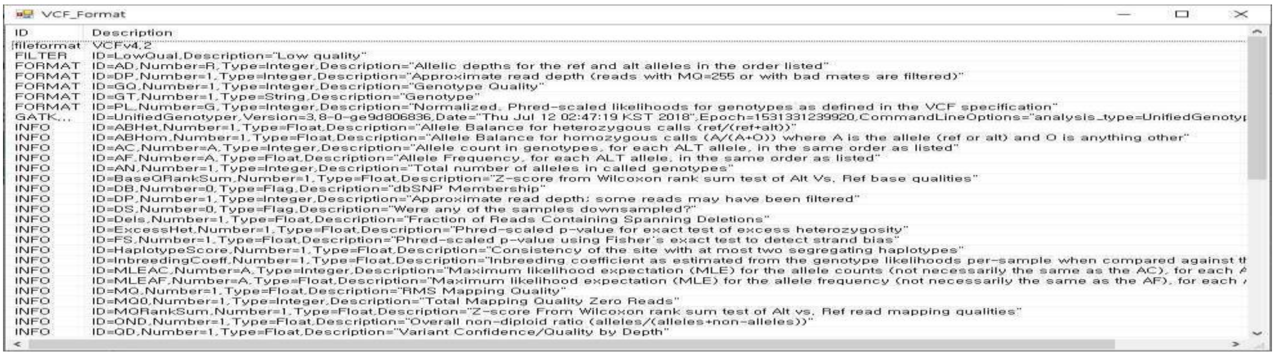

Figure 11. VCF Format Information Window

(6) VCF Filter Panel

The VCF Filter Panel is utilized to filter the output variant information in the Exon Variant Call area (See panel (2) in the upper part of Figure 10). Depending on the variant types, a user can selectively output the variants and designate an allele frequency value to selectively output only the variants not exceeding the designated value. After setting all the conditions for filtering, click the 'Reload' button to output the filtering results in the Exon Variant Call area.

(7) Information Panel

The Information Panel displays the name, location of a gene and transcript under a running browsing (See Figure 12). The gene or transcript name selected in the Gene/Transcript View area is shown. The location of exon including the variant selected in the Exon Variant Call Panel is shown. In addition, the location of mouse pointer in the Main Exon View Panel is also displayed.

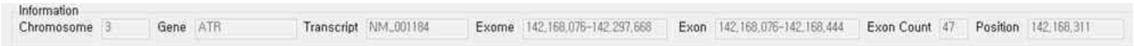

Figure 12. Information Panel

(8) Main Exon View Panel

Select and click a random variant in the Exon Variant Call Panel to output detailed information on exon area including the variant in the Main Exon View Panel (See Figure 13). The Main View Panel area consists of 3 different content areas having respective functions (See Table 6);

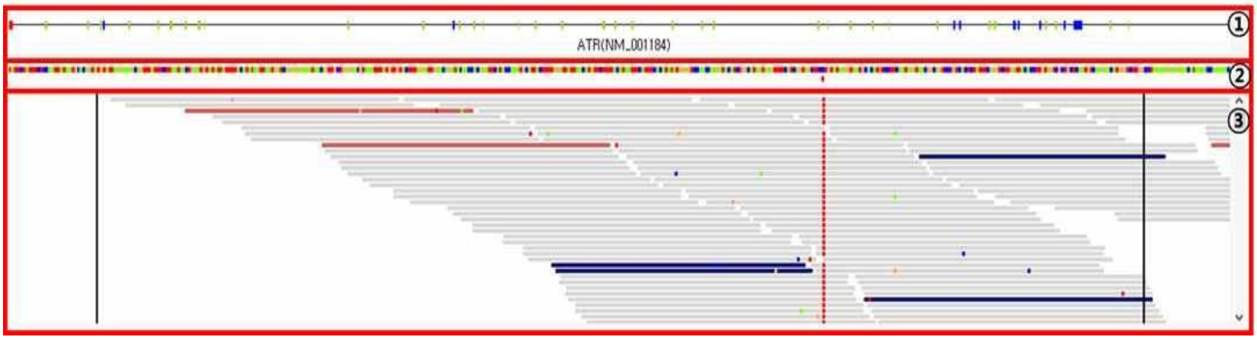

Figure 13. Main Exon View Panel

<Table 6> Functions of the Main Exon View Panel's content Areas

No.	Content	Function
①	Transcript structure viewer	Displays the exon-intron structure of a transcript including a variant of interest.
②	Variants along the reference sequence	Displays the location of a variant on the reference sequence.
③	Read alignment viewer	Outputs the read alignment result on an exon area including a variant of interest.

① Transcript structure viewer

This shows the exon-intron structure of a transcript including the variant of interest. The exon and intron areas are each marked with square box and line, respectively. The square box representing the exon area is output in 3 different colors: green, blue and red (See Figure 14). An exon area without a variant is green, with a variant is blue, and which is displayed as a current view in the Read alignment viewer including the variant of interest in red. If the mouse pointer is placed on a square box which represents an exon area, the system shows information about that corresponding exon such as its serial number and position (See Figure 15). In the exon-intron structure of a transcript, a user can move to a random exon area by mouse click. With such move, the "Variants along with the reference sequence" panel and "Read alignment viewer" panel become synchronized and indicate the information about the exon area of interest.

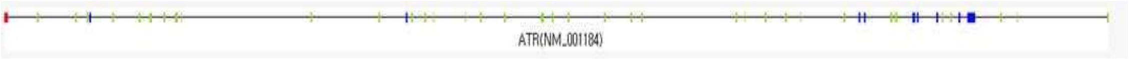

Figure 14. Transcript Structure Viewer

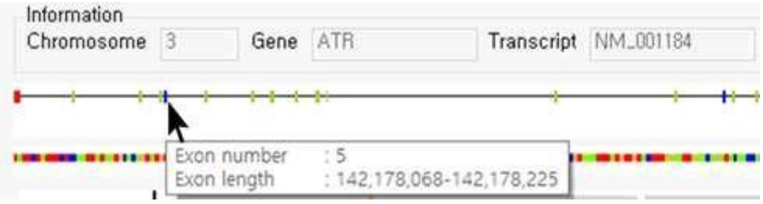

Figure 15. Example of Exon Area Information Output

② Variants along the reference sequence

A variant called from an exon area is output in the lower part of the reference sequence (See Figure 16). Each base of the reference sequence is marked distinctively in different colors (See Table 7). The variant shown in the lower part of the reference sequence also shows base types in different colors. When a mouse cursor is brought to a random position of the reference sequence, the system shows the accurate location in the genomic space in the Position Information inside the Information Panel.

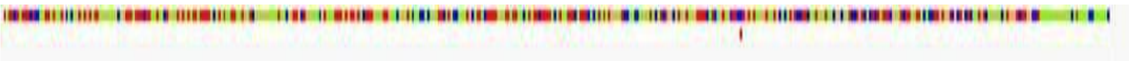

Figure 16. Variant Positions along the Reference Sequence

<Table 7> Colors of different Reference Sequence Bases

Color	Base
Lawn Green	A
Blue	C
Sandy Brown	G
Red	T
Gray	N

③ Read alignment viewer

The read alignment results of an exon area are output together with diverse data (See Figure 17). The main specifications are as follows.

▶ Scope of the genomic area to output: The basic genomic area which is displayed includes an exon area and a part of the intron area in its upstream and downstream. In Figure 17, the outside of the black-lined area denotes an intron area neighboring the exon area, and the default values of 30 bases in the upstream and downstream of the exon area is included in the basic output scope. The scope of the intron area output, however, is adjustable in the Viewer Control Panel.

If the coverage depth of aligned read sequences is too deep to output read alignment results on single monitor page, a user can check more read alignment results in the upper and lower sides by using a mouse wheel or scroll bar. Double click a random aligned read sequence to call the Genome Browser and analyze in-depth the read alignment results in the genomic area centered at the related position.

▶ View read alignment results: In the read alignment results, if a read sequence perfectly matched to the reference sequence, each base of the read sequence becomes colored in light gray. If there are mismatches, the read sequence bases are colored differently according to their types (See Table 7). Each read sequence of paired-end reads has its own direction. The forward read starts from the left side and ends on the right side. The reverse read starts from the right side and ends on the left side. To indicate the direction of the aligned reads, an arrow is given to the base at the end of each read. Moreover, to differentiate the insert size of the aligned paired-end reads, the reads with insert size larger than the max value (default = 400) are colored red; the ones with insert size smaller than the min value (default = 100) are blue, and those between the max and min values are light gray. The

max/min value of the insert size is adjustable in the Exon View Control Panel.

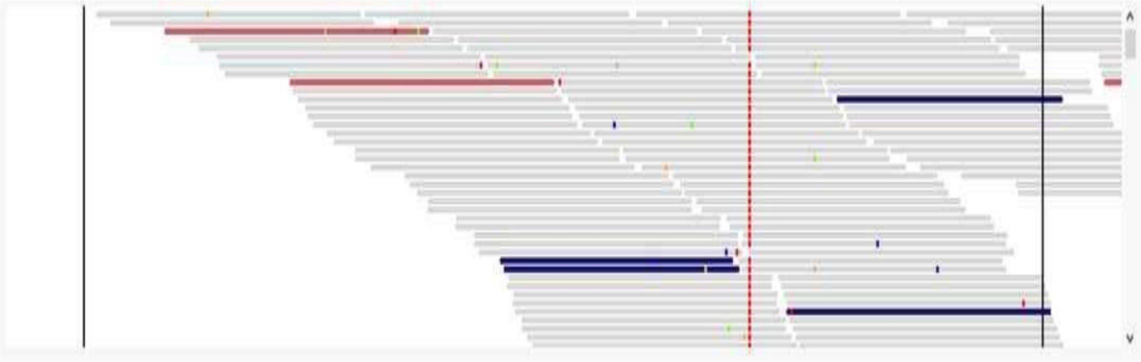

Figure 17. The Read Alignment Viewer

▶ Variant calling result output: The SNP, insertion, and deletion variant information are output in the 'Read alignment viewer' in the following manner.

▶▶ SNPs: If the base of the read sequence does not correspond to the reference sequence base in the read alignment results, the read sequence base is marked differently according to base types and not in light gray (See Table 7). Therefore, in the event of an SNP variant in the read alignment results, the read sequence bases are marked in a color other than light gray and can be visually confirmed. In Figure 18, the area in the red box represents an example of an SNP variant appearance. If the mouse cursor is brought to that position, the system outputs the accurate genomic position in the Position box inside the Information panel.

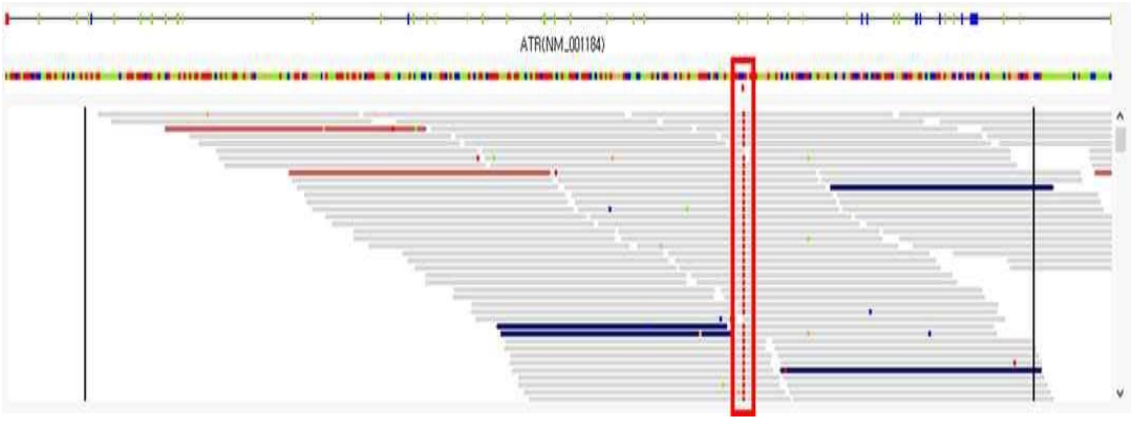

Figure 18. Example of SNP Variant Area Indication

▶▶ Deletions: If any deletion variant is detected in the read alignment results, compared with the reference sequence, the related base sequence, which is a part of the read sequence, is indicated by a black horizontal bar. Therefore, in the event of the presence of a deletion variant in the read alignment results, a user can visually check the base sequence part in the variant position, which is marked by a black bar instead of light gray. In Figure 19, the area in the red box represents an example of a deletion variant appearance. If the mouse cursor is brought to that position, the system outputs the accurate genomic position in the Position box inside the Information panel.

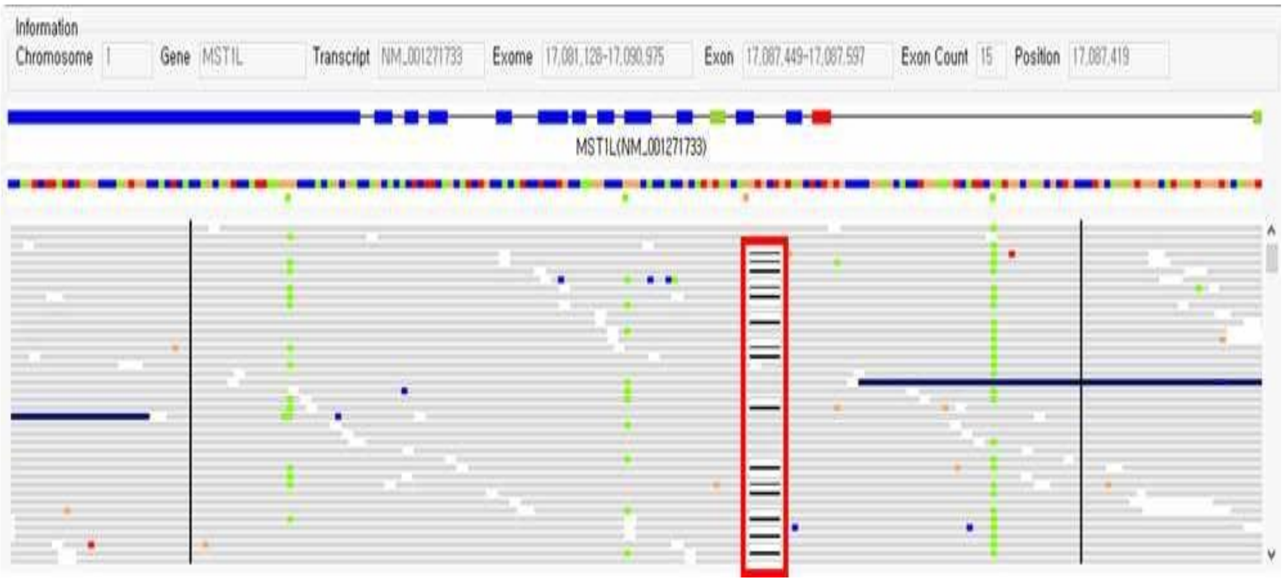

Figure 19. Example of Deletions Variant Area Indication

▶▶ Insertions: In the event of an insertion variant detected in the read alignment results, compared with the reference sequence, a purple box is inserted to indicate the position of the read sequence base. In Figure 20, an example of the insertion variant is shown in the place indicated by the mouse pointer. However, since the position of the insertion variant appearance is marked between the base characters, it may be hard to visually distinguish in the Read alignment viewer. In such a case, double click the mouse in that position and call the Genome Browser to look at the insertion variant area more closely (See Figure 21).

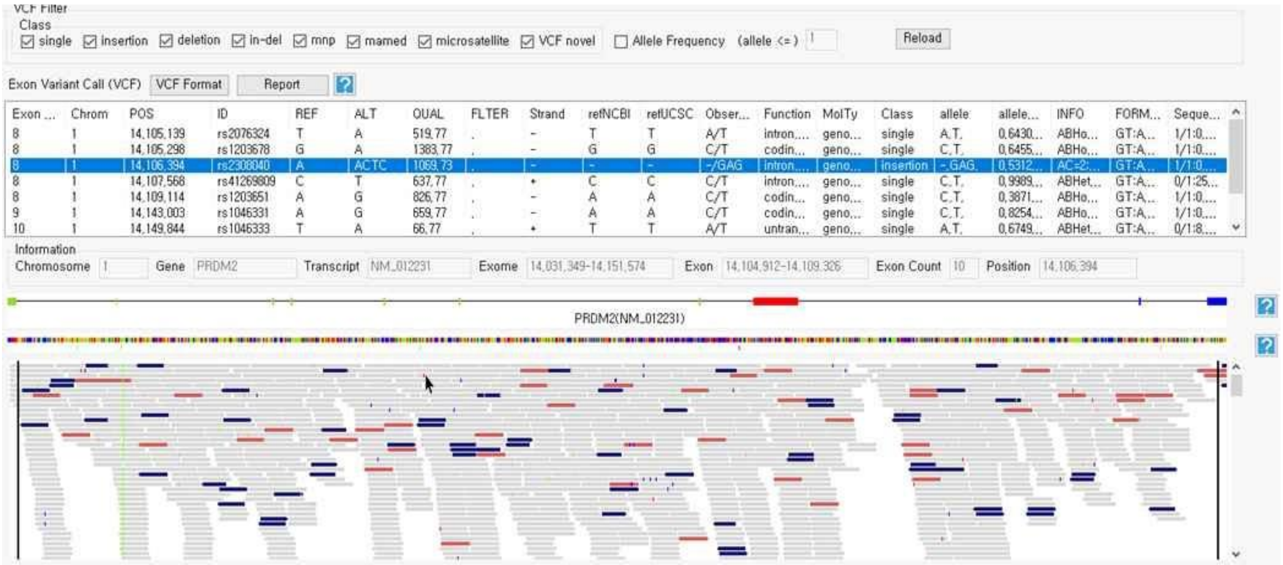

Figure 20. Example of Insertion Area Indication

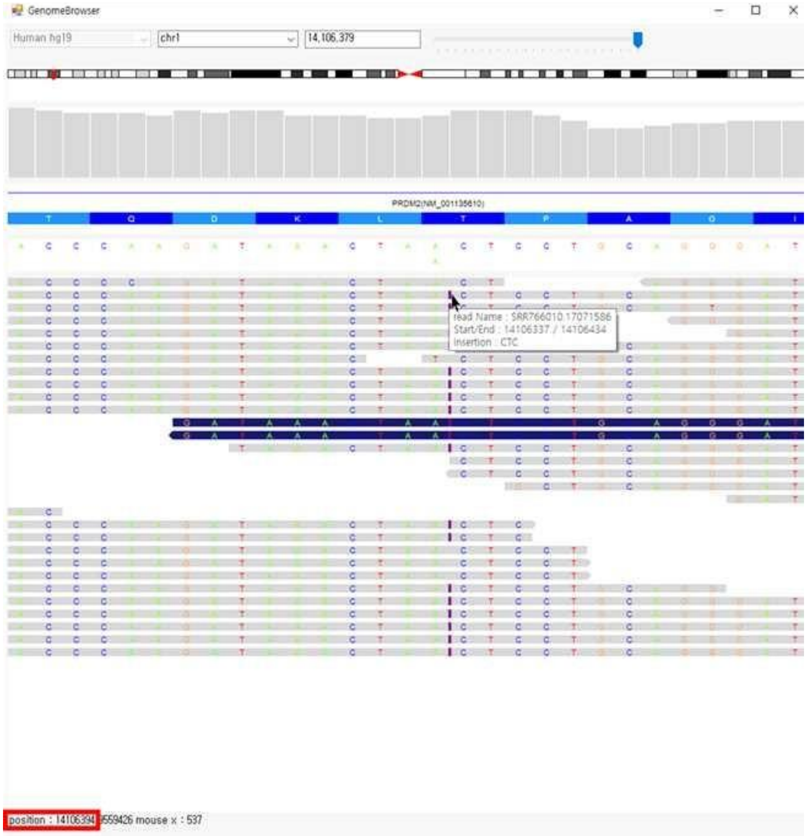

Figure 21. Example of Insertion Variant Area Indication in Genome Browser

(9) Exon View Control Panel

This is utilized to control the Main Exon View Panel (See Figure 22). Its functions are categorized into four groups as exhibited in Table 8.

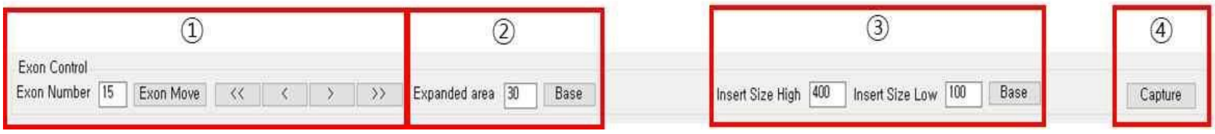

Figure 22. The Exon View Control Panel

<Table 8> Functions of the Exon View Control Panel

Content	Function
Exon Number [1] Exon Move << < > >><br>Exon Move Controller	Move in exon areas.
Expanded area [30] Base<br>Intron Area Select Controller	Sets the scope of the intron area to be included in the basic output area.
Insert Size High [400] Insert Size Low [100] Base<br>Insert size Controller	Sets the minimum and maximum insert size of paired-end reads.

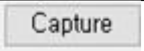 Capture button	Panel capturing

① Exon Move Controller

Enter an exon number in a text box to set an exon number in the Exon Move Controller, and click Exon Move to move to an exon area of interest. The exon number indicates the number of consecutive indexing of the exons in a transcript according to the genomic position. Furthermore, a user can move between the exon areas using a << < > >> button. The << >> button moves between 5 exon numbers at a time, and the < > button moves between single exon numbers.

② Intron Area Select Controller

This controller is utilized to adjust the size of intron areas in the upstream and downstream of an exon displayed in the Read alignment viewer. The initial value is set at 30, and the intron areas in the range of 30 bases in the upstream and downstream of the exon are presented as output. For adjustment, the whole numbers can be utilized within the adjustable range from 0 to 200.

③ Insert size controller

The Insert size controller is utilized to adjust the min/max values of insert size to differentiate read alignment results with colors depending upon the insert size of paired-end reads. The initial min/max values are set at 100/400, respectively. For adjustment, the whole numbers can be utilized within the adjustable range from 0 to 99.99.

④ Capture button

The Capture button captures the present output contents of the Main Exon View Panel and Information Panel and stores them as images in the jpg format (See Figure 23).

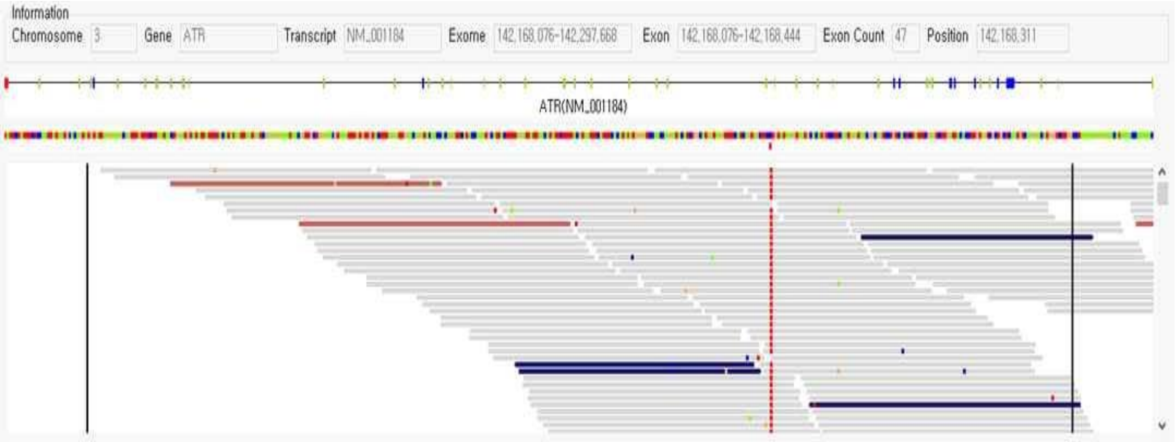

Figure 23. Example of a Captured Image using Capture Button
