# Supplementary material for: PhenGenVar: A User-Friendly Genetic Variant Detection and Visualization Tool for Precision Medicine
Source: J Pers Med. 2022 Jun 12;12(6):959. doi: 10.3390/jpm12060959 (PMC9224645; doi:10.3390/jpm12060959)
Supplement: Supplementary file 1 [file jpm-12-00959-s001.zip › PhenGenVar_Genome_Browser manual.pdf]

Genome Browser Manual

The Genome Browser is called from the Exome Browser and utilized to closely browse the variant calling results based on read alignment. However, before using the Genome browser, the read alignment file (BAM format) must be loaded through the Exome Browser. A user can selectively input a variant calling file (VCF format). The Genome Browser can be called using any of the following three ways for execution.

i) To open the Genome browser, select it from the Menu Bar of the Exome Browser (See Figure 1).

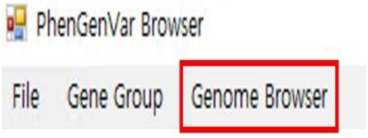

Figure 1. How to call Genome Browser through Exome Browser's Menu Bar

ii) The Genome browser can also be called by selecting and double clicking a certain variant record in the Exon Variant Call Panel of the Exome Browser (See Figure 2). Since the Genome Browser is called from the genomic position of a variant, the genomic areas in the front and back of that position are loaded in the Browser. Figure 3 exhibits the execution page of Genome Browser called by double clicking the variant record selected in Figure 2. Additionally, various optional functions are offered for more in-depth search of the read alignment results centering on the selected variant position.

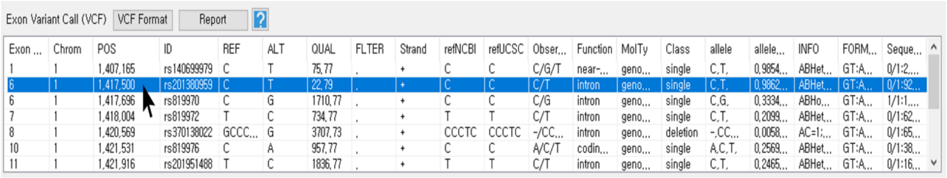
Exon Variant Call (VCF) VCF Format Report

Exon ...	Chrom	POS	ID	REF	ALT	QUAL	FLTER	Strand	refNCBI	refUCSC	Obser...	Function	MolTy	Class	allele	allele...	INFO	FORM...	Seque...
1	1	1,407,165	rs140699979	C	T	75,77	.	+	C	C	C/G/T	near-...	geno...	single	C,T,	0,9854...	ABHet...	GT:A...	0/1:2,...
6	1	1,417,500	rs201380959	C	T	22,79	.	+	C	C	C/T	intron	geno...	single	C,T,	0,9862...	ABHet...	GT:A...	0/1:92...
6	1	1,417,696	rs819970	C	G	1710,77	.	+	C	C	C/G	intron	geno...	single	C,G,	0,3334...	ABHo...	GT:A...	1/1:1,...
7	1	1,418,004	rs819972	T	C	734,77	.	+	T	T	C/T	intron	geno...	single	C,T,	0,2099...	ABHet...	GT:A...	0/1:62...
8	1	1,420,569	rs370138022	GCCC...	G	3707,73	.	+	CCCTC	CCCTC	-/CC...	intron	geno...	deletion	-,CC...	0,0058...	AC=1;...	GT:A...	0/1:65...
10	1	1,421,531	rs819976	C	A	957,77	.	+	C	C	A/C/T	codin...	geno...	single	A,C,T,	0,2569...	ABHet...	GT:A...	0/1:38...
11	1	1,421,916	rs201951488	T	C	1836,77	.	+	T	T	C/T	intron	geno...	single	C,T,	0,2465...	ABHet...	GT:A...	0/1:16...

Figure 2. How to call Genome Browser in Exon Variant Call Panel

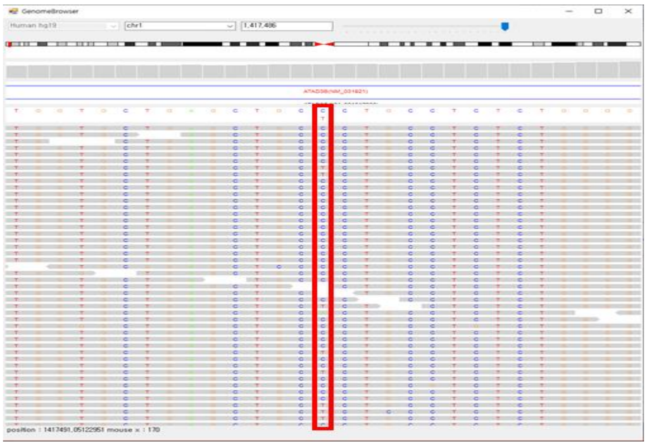

Figure 3. Example of execution page of Genome Browser called from the Exon Variant Call Panel

iii) The third way to open the Genome browser is to double click in the Read alignment viewer area or Variants along the reference sequence area of the Exome Browser's Main Exon View Panel (See Figure 4). Since the Genome Browser is called from the position of a genome, the genomic areas in the front and back of that particular position are loaded in the browser.

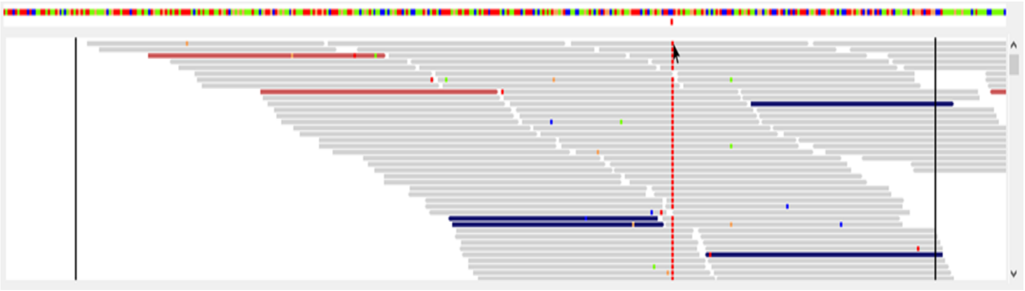

Figure 4. How to call Genome Browser from the Exome Browser's Main Exon View Panel

The Genome Browser's main page consists of 5 areas (See Figure 5) and the functions of each area are listed in Table 1.

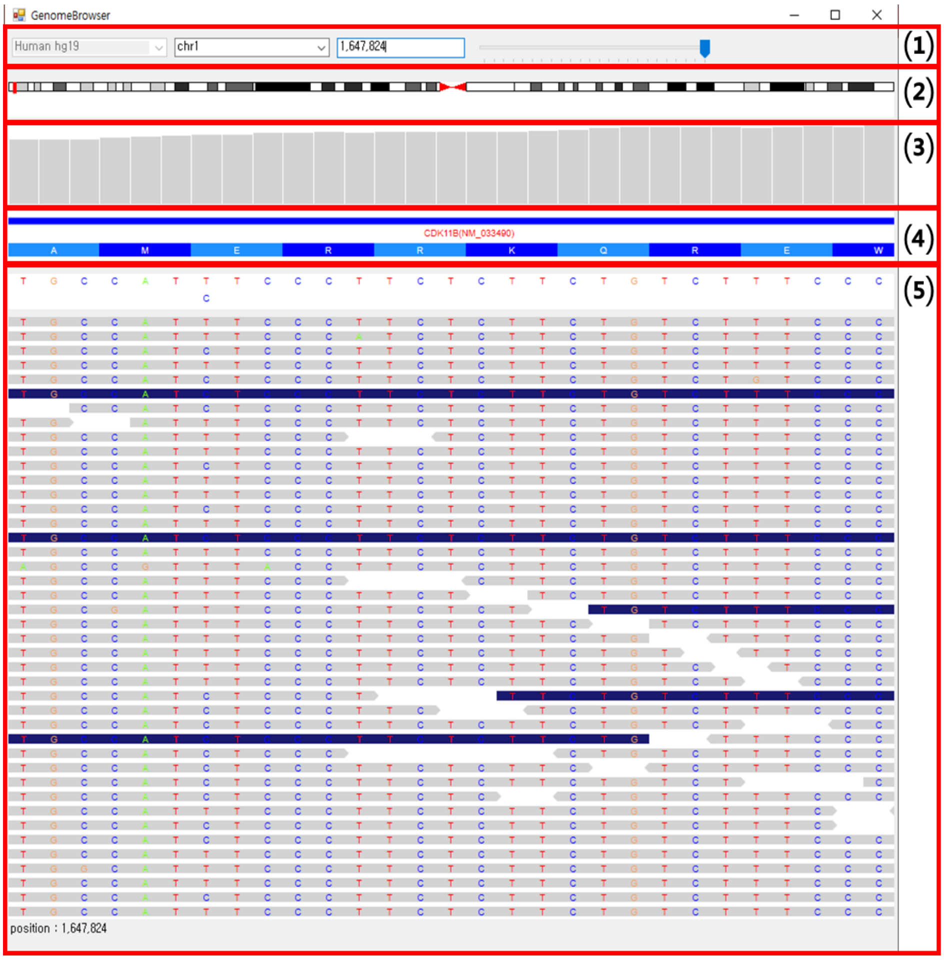

Figure 5. The Genome Browser's Main Window

<Table 1> Genome Browser's Page Contents & their Functions

No.	Contents	Function
(1)	Control Panel	Sets up the genomic position in reference sequence and resolution level for browsing.
(2)	Cytoband Panel	Cytoband representation
(3)	Coverage graph Panel	Read depth representation
(4)	Genetic structure Panel	Genetic structure indication
(5)	Main Genome View Panel	Shows read alignment results and variant positions in reference sequence.

(1) Control Panel

The Control Panel is data/configuration setup panel for BAM/VCF file analysis and consists of 4 areas (See Figure 6) with respective functions, as shown in Table 2;

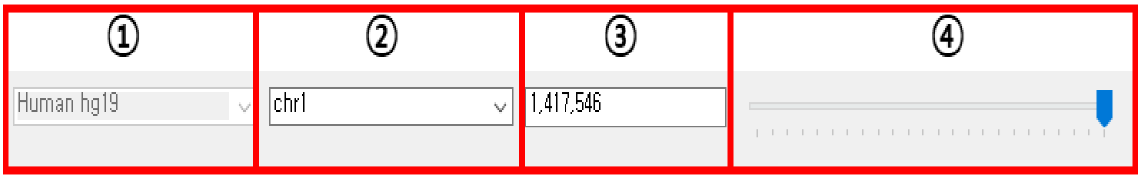

Figure 6. Contents of the Control Panel

<Table 2> Control Panel's Page Contents & Functions

No.	Contents	Function
①	Reference sequence combo box 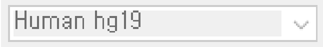	Shows the reference genome sequence version.
②	Chromosome combo box 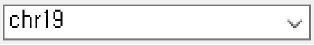	Selects the chromosome number.
③	Search text box 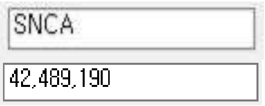	Inputs a gene name or genomic position.
④	Track Bar 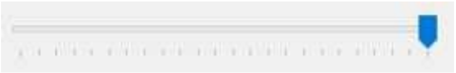	Sets the resolution level.

① Reference sequence combo box

This box shows the reference genome sequence version selected in the Genome Browser. The sequence version is not changeable. The browser supports the reference sequence versions of Human hg_19 and Human hg_38.

② Chromosome combo box

This box shows the chromosome number set up in the Genome Browser. The value is changeable. In the event of making a call without a chromosome number in the Exome browser, the chromosome number is set to 1 as default.

③ Search text box

This box is utilized to set a genomic position on a chromosome. The input position is set at the center of each panel. Position information is randomly changeable. A user can key in a gene name firsthand to set the position of a gene of interest at the panel center. During browser operation, however, the head position of each panel is displayed.

④ TrackBar

The Trackbar is used to increase or decrease the resolution of each panel screen. By adjusting the TrackBar, every panel monitor of the Genome Browser is enlarged/reduced with respect to the central position. The resolution is calculated by the following equation;

$$S = \frac{ChrLength}{2^n}$$

Where, n represents a resolution level, and the range of n values is from 0 to 22. S represents the max length of the genomic area in the nucleotide base-pair (bp) unit, which is output in the monitor according to the resolution level (n value), ChrLength signifies the length of a chromosome. As an example, in the read alignment viewer, for chromosome number 1, the output of genomic area in a single panel is 121,704bp at the min resolution and 59bp at the max resolution.

(2) Cytoband Panel

This panel shows the cytoband of chromosome which is being browsed. Click a random position on cytoband indication with a mouse to set the position as the central position of all the panels and start browsing. The genomic area of the ongoing browsing in the present panel is indicated by red box in the cytoband (See Figure 7).

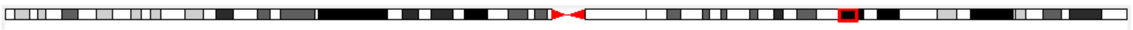

Figure 7. The Cytoband Panel

(3) Coverage graph Panel

The dynamic calculation of read depth for each genomic position is done from read alignment files (BAM file) and the calculated results are output through a coverage graph in the Coverage graph panel. The resolution of the coverage graph can be adjusted using the Control Panel's

TrackBar (See Figure 8). If the mouse pointer is brought to a random position in a coverage graph, the system shows the genomic position and read depth in numbers (See Figure 9).

Figure 8. Example of a Coverage Graph

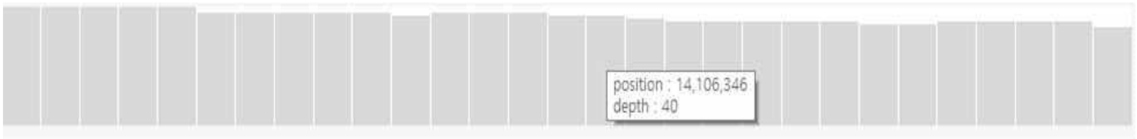

Figure 9. Detailed Data Indication through Mouseover on the Coverage Graph

(4) Genetic structure Panel

The Genetic structure Panel shows the structure of a gene in a genomic area and displays the gene name and detailed exon-intro structure of the registered transcripts. On opening the Genome Browser by double clicking a variant record in the Exome Browser's Exon Variant Call Panel, the information about the gene (transcript) including the variant of interest is displayed in the Genetic structure Panel. Especially, the gene name/transcript name is displayed in red for easy identification, while the other gene/transcript names are all colored in black (See Figure 10). If the browser resolution is set low, such gene/transcript's exon-intron structure is output. On the other hand, at high resolution and magnified screen, the amino acid sequence of the gene/transcript is output (See Figure 11). The amino acid sequence is marked with a square colored alternatively in blue and sky-blue. The Exon areas are marked with thick lines, while the intron areas with thin lines (See Figure 11). When multiple transcripts are reported for a single gene, a user can search them by moving up and down the panel area using a mouse wheel.

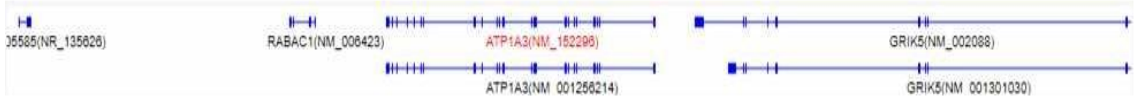

Figure 10. Gene (Transcript) Structure Indication at Low Resolution

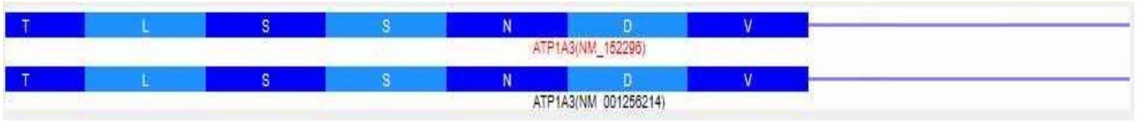

Figure 11. Example of Amino Acid Sequence Indication at High Resolution

(5) Main Genome View Panel

The Read alignment and variant calling results are output together with diverse information (See Figure 12). The Main View Panel area is composed of 2 areas whose main functions are as follows (See Table 3);

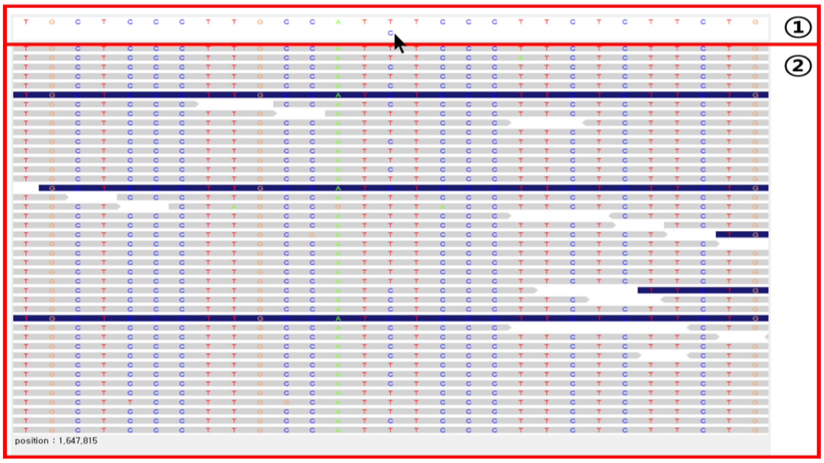

Figure 12. The Main Genome View Panel

<Table 3> Functions of Each component Area of the Main Genome View Panel

No.	Composition	Function
①	Variants along the reference sequence	Shows a variant position in reference sequence.
②	Read alignment viewer	Outputs read alignment results.

① Variants along the reference sequence area

A variant reported in a variant calling file (VCF format) is output in the lower part of the reference sequence. Each base of the reference sequence is indicated by different colors (See Table 4). The base type of a variant displayed in the lower part of the reference sequence is also distinguished by different colors. Mouseover a random position of the reference sequence to output a genomic position in the position information in the browser's bottom part. Information on the base type is output in colored bars in the event of reduction to the minimum size and colored letters in the event of maximum enlargement (See Figure 13-(a), 13-(b)).

<Table 4> Color Indication of Reference Sequence Bases

Color	Base
Lawn Green	A (adenine)
Blue	C (cytosine)
Sandy Brown	G (guanine)
Red	T (thymine)
Gray	N

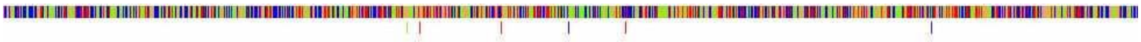
Figure 13-(a) Reduction to the Minimum

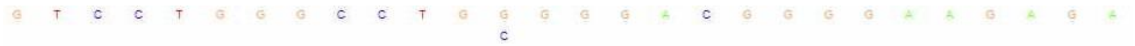

Figure 13-(b) Enlargement to the Maximum

② Read alignment view area
This shows the detailed information of each read aligned in the reference sequence and read mapping data by using various visualization methods. It displays the variant calling information inferred from the read alignment results, together with additional data. In the browser area of default output, a user can move an indication area through mouse drag. Drag the mouse to the left to move to the previous position of the read alignment result presently output, whereas drag the screen to the right to move to the position next to the presently-displayed read alignment result. If the coverage depth of the output reads is too deep to display the read alignment results in a single page, roll the mouse wheel to move up and down. The Read alignment viewer has the following main functions.

▶ Read realignment: The read alignment results in the browser are basically based upon the read alignment data of the input BAM files. This browser additionally employs the CIGAR data of the BAM files to implement read realignment. The purpose of read realignment is to correctly rearrange the reads in the reference sequence. Mouseover each of the aligned reads to output detailed data of each read.

▶ Read alignment result enlargement and reduction: A user can enlarge or reduce read alignment results by adjusting the resolution level through the Control Panel's TrackBar. At higher resolution, each base of the read sequence and reference sequence is indicated with different colors according to the base type (See Table 4 and Figure 14). However, if the resolution is lower, and the base of a read sequence coincides with that of the reference sequence, each base of the read sequence is colored light gray. If they do not coincide, only the base of the read sequence is distinguished by color depending upon the base type (See Figure 15).

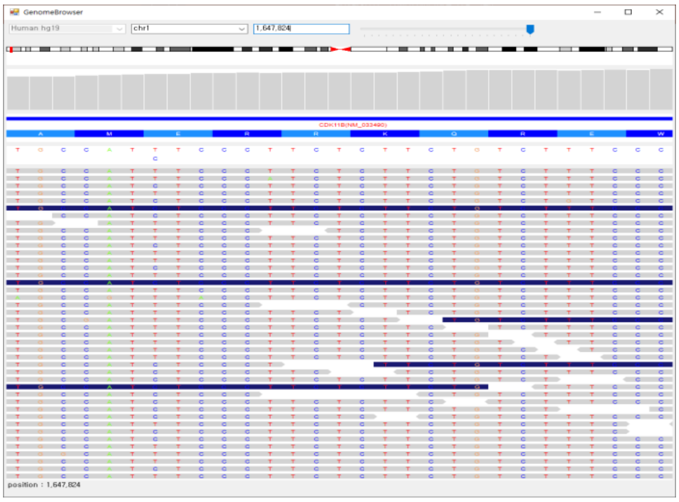

Figure 14. View of an Enlarged Read Alignment Result

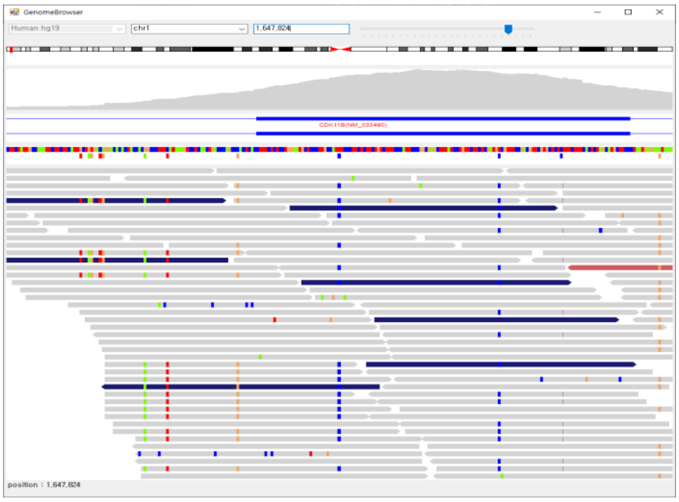

Figure 15. View of a Reduced Read Alignment Result

▶ Read direction indication: Each read of paired-end reads has a direction. A forward read starts from the left side and ends at the right, whereas a reverse read starts from the right and end at the left. To indicate the direction of aligned reads, an arrow is provided to the end base of each read (See Figure 16).

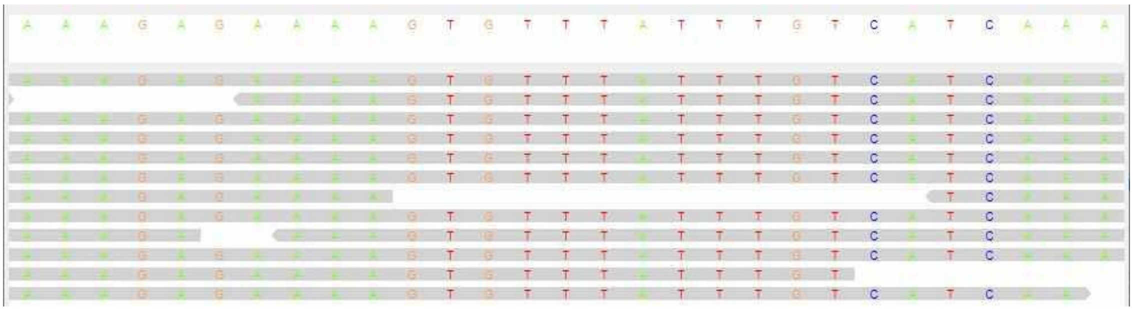

Figure 16. Read Direction Indication

▶ Reads insert size indication: To distinguish the insert size of aligned paired-end reads, the background of read sequence is differently colored. For reads with insert size larger than the max value (default = 400), the read sequence background is red; and for reads smaller than the min value (default = 100), the background is blue. Those between the max and min values are gray in color (See Figure 17). The max/min value of insert size can be adjusted from the Exome Browser's Exon View Control Panel.

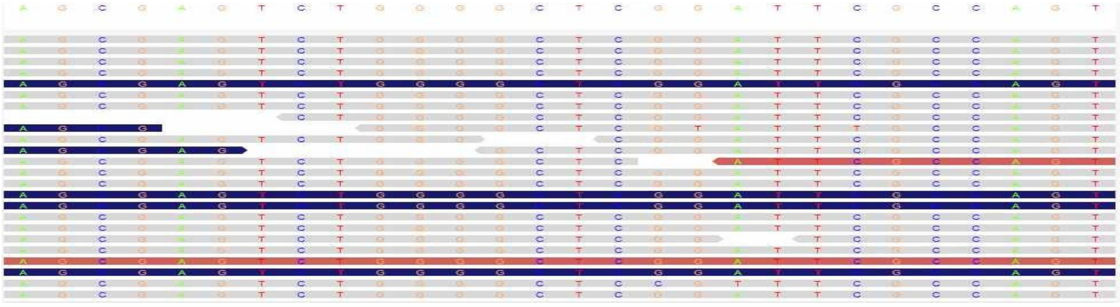

Figure 17. Reads Insert Size Indication

▶ Variant calling results: The results include SNP, insertion, and deletion information which are output in the Read alignment viewer in the following manner.

▶▶ SNPs: In the read alignment analysis, if the base of a read sequence does not coincide with the reference sequence base, they are differentiated in different colors according to the base type (See Table 4). Therefore, for an SNP variant in the read alignment results, bases marked in different colors from the reference sequence bases are visually confirmed in the involved SNP position (See Figure 18). In Figure 18 below, the area inside the red box represents an example of an SNP variant appearance. If the mouse cursor is brought to the position, the system outputs the genomic position in the Position information panel located in the lower left part of the browser. On the other hand, if a variant information file (VCF file) input by a user is loaded, and the related SNP data are included, the called variant in the Variants along the reference sequence area is also output together in the bottom part of the reference sequence (See Figure 19).

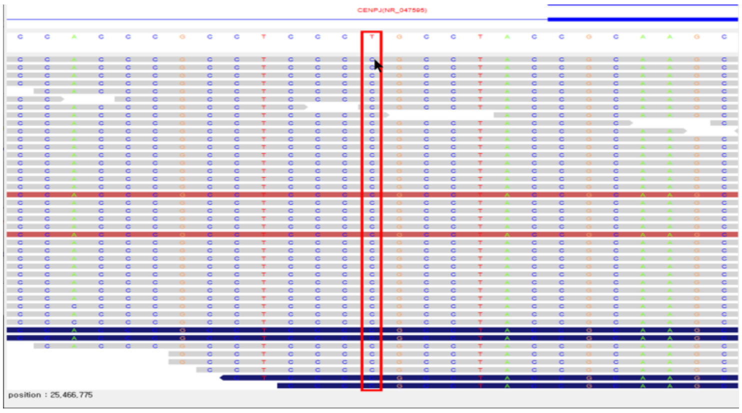

Figure 18. View of an SNP Variant Area Indication

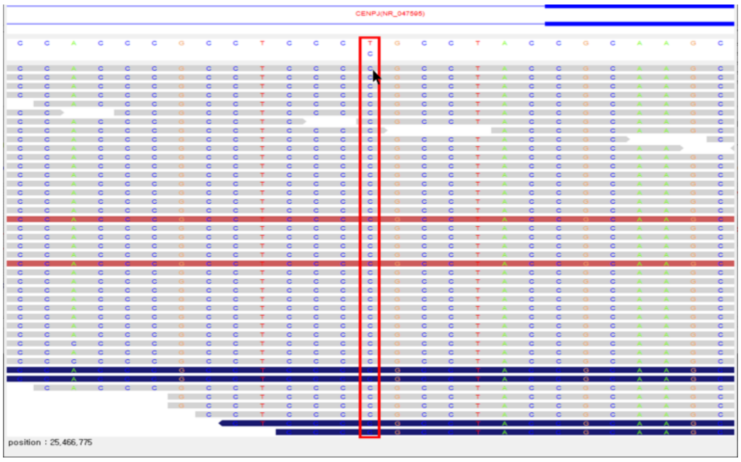

Figure 19. View of an SNP Variant Area Indication with Loaded VCF File Data

▶▶ Deletions: When a deletion variant is detected in the read alignment analysis, the base sequence part of the related read sequence is indicated with a black horizontal bar (See Figure 20). Therefore, when a deletion variant is present in the read alignment results, a user can visually check the base sequence part marked with a black bar at that variant's position. In Figure 20, the area under the red box shows an example of the emergence of a deletion variant. If a user brings the mouse cursor to that position, the system outputs the genomic position in the Position information panel in the lower left part of the browser.

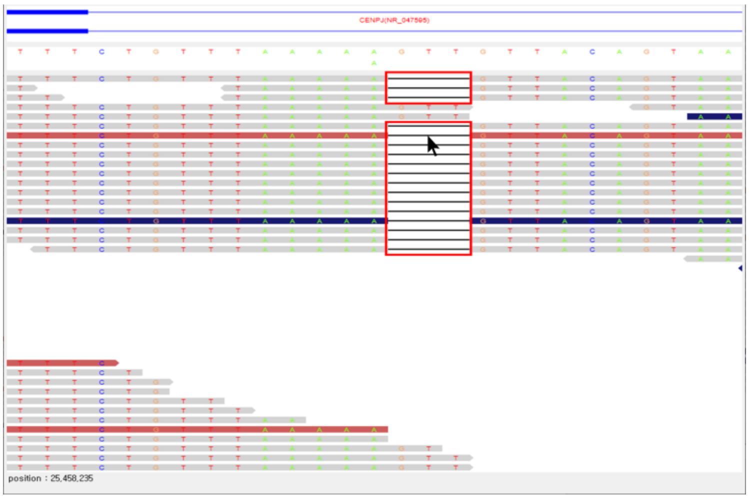

Figure 30. View of a Deletion Area Indication

▶▶ Insertions

When an insertion variant is detected in the read alignment analysis, a purple box appears as a notification at the position of the base of the read sequence (See Figure 21). Figure 21 shows the appearance of insertion variants in the red box area. If a mouse cursor is brought to the position where an insertion variant is found, the system outputs detailed information about the insertion variant, such as the number of inserted bases and base types.

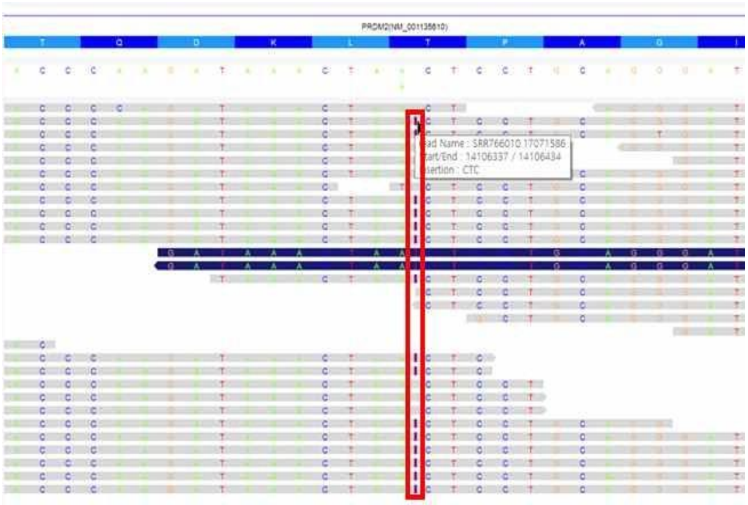

Figure 21. View of an Insertion Area Indication
