# Supplementary material for: PhenGenVar: A User-Friendly Genetic Variant Detection and Visualization Tool for Precision Medicine
Source: J Pers Med. 2022 Jun 12;12(6):959. doi: 10.3390/jpm12060959 (PMC9224645; doi:10.3390/jpm12060959)
Supplement: Supplementary file 1 [file jpm-12-00959-s001.zip › Supplementary Figure S1.pdf]

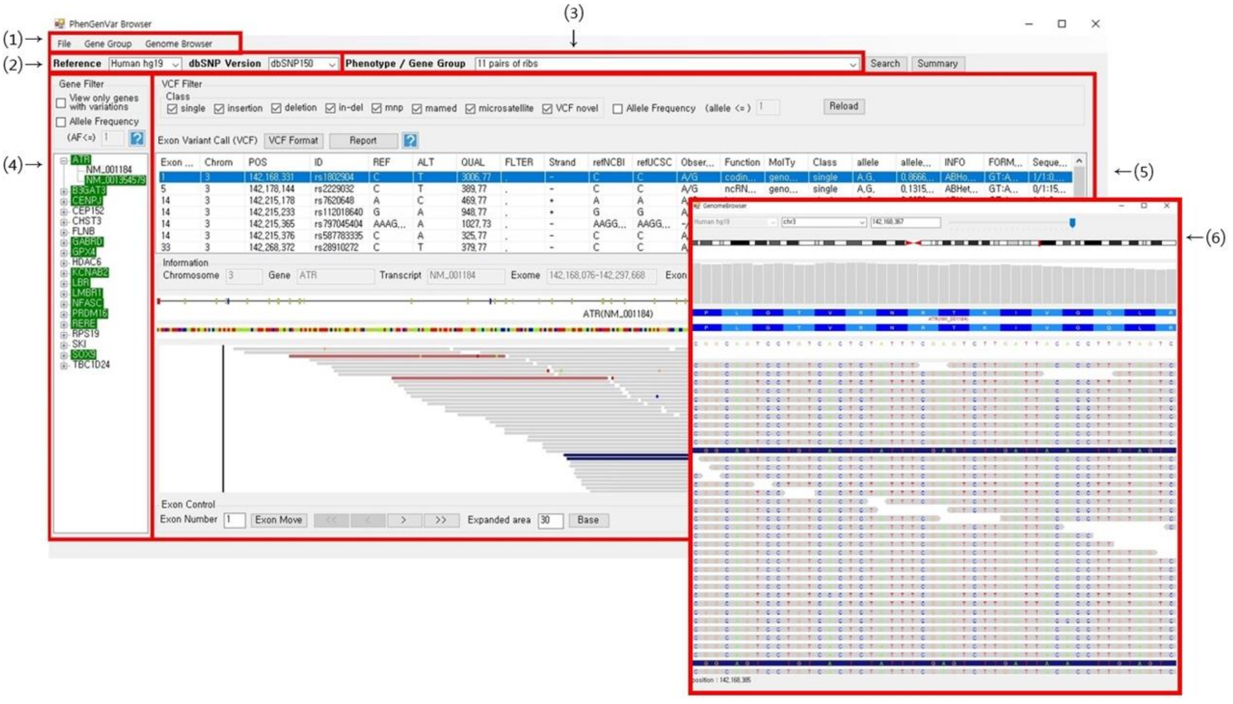

Supplementary Figure S1. Simple steps to use PhenGenVar Browser.
(1) Load a BAM file and a VCF file containing variant calling results (optional) by selecting them using the File menu. Note: select only BAM and VCF files that have index files (*.bai/*.idx). (2) Select a reference sequence version and a dbSNP version (optional) which are necessary for BAM/VCF file analysis. (3) Select a Gene_Group or Phenotype to analyze. The related gene sets are displayed in the Gene/Transcript View area. (4) Double click a random gene output in the Gene/Transcript View area to obtain the transcript data of the gene in a child node. If a random transcript is double-clicked, the variant information areas are activated to provide the variant list identified on the transcript of interest. (5) Select and click a random variant to display an exon area including the position of the selected variant in the Main Exon View Panel. (6) Select a variant and double click with a mouse to call a Genome Browser centering at the position of the related variant and use it for a detailed analysis of that area.
